# Beyond Single Descriptors: Complementary Feature Learning for Image Matching

**DOI:** 10.3390/jimaging12050201

**Published:** 2026-05-05

**Authors:** Xianguo Yu, Yulong Feng, Xi Li

**Affiliations:** 1 College of Intelligence Science and Technology, National University of Defense Technology, Changsha 410073, China; yuxianguo11@nudt.edu.cn; 2 National Key Laboratory of Equipment State Sensing and Smart Support, National University of Defense Technology, Changsha 410073, China; 3 Hunan Provincial Key Laboratory of Flexible Electronic Materials Genome Engineering, Changsha University of Science and Technology, Changsha 410076, China

**Keywords:** local feature matching, complementary descriptors, orthogonal loss, pose estimation

## Abstract

Sparse local feature matching has served as the cornerstone of numerous visual geometry tasks and attracted extensive attention. Although significant progress has been made in this area, improving the discriminative power of descriptors remains a key challenge. As far as we know, existing sparse feature matching methods only predict a single descriptor map for keypoints, which might restrict their potential in solving complex scenarios. This issue is particularly pronounced in real-time applications where most methods only learn descriptor maps at a reduced spatial resolution compared to the input image. Consequently, they require interpolating from the low resolution map for obtaining per-keypoint descriptors, which will introduce background contamination and reduce the discriminability of final descriptors. To address these issues, we propose an efficient novel complementary local feature description model. Specifically, the model simultaneously learns two descriptor maps using different loss functions within a single Convolutional Neural Network (CNN). An orthogonal loss is introduced to effectively coordinate the learning of the two branches, aiming to obtain decoupled and complementary descriptors. Extensive experiments across various visual geometry tasks, such as homography estimation, indoor and outdoor pose estimation, as well as visual localization, have demonstrated the superior performance of the proposed method.

## 1. Introduction

Local feature matching constitutes a fundamental block for 3D computer vision applications such as Structure from Motion (SFM) [1], Simultaneous Localization and Mapping (SLAM) [2,3], and visual localization [4]. Current learning-based methodologies for this task are generally divided into two categories. One is the sparse matching approaches [5,6] which employ explicit feature detectors and perform image matching based on them. The other one is the dense matching technique [7] which directly predicts matches without localizing local keypoints.

The dense matching approaches accept a pair of images as input and output their matches in a forward pass. They often employ stacked transformer blocks with self-attention and cross-attention mechanisms to learn mutual information between the matching images, leading to substantial computational overhead. In contrast, the sparse matching methods are more efficient and preferred for real-time applications. Generally, the sparse matching paradigm [8] usually follows a three-stage workflow. Firstly, the feature detection stage predicts a score map and identifies keypoints through non-maximum suppression. The subsequent feature description stage then extracts keypoint-wise descriptors by sampling from learned high-dimensional feature maps. Finally, the matching stage establishes correspondences by computing similarities between different descriptors and generates matches according to the nearest-neighbor principle.

Prevailing sparse matching researches [5,9,10] predominantly learn only one descriptor for each keypoint by minimizing the distances between matched features while maximizing the distances between negative pairs. Such a strategy may limit their capabilities in complex scenarios because a single descriptor might not fully capture the diverse characteristics required to handle intricate situations. In this paper, a comparative experiment is conducted by using SuperPoint [5] as the same keypoint detector and leveraging different feature descriptors for image matching. It turns out that there is minor performance gap between different feature descriptors, as shown in Table 1. The experimental results have driven us to learn and leverage multiple descriptors simultaneously for robust image matching.

Furthermore, the real-time applications have thrown a new challenge to image matching algorithms. To reduce computational complexity, most methods predict low-resolution feature maps and output pixel-wise keypoint descriptors by bilinear interpolation from them. Since the interpolation process takes into account neighboring feature descriptors, the discriminative power of each keypoint descriptor can be compromised. In this paper, we learn two feature descriptors for each keypoint to alleviate this problem.

Though researchers have tried to ensemble different matching methods to improve image matching performance [11], to the best of our knowledge there is no prior work that combines different feature descriptors for keypoint appearance representation. As we will demonstrate in Section 4.5, simply ensembling descriptors from different models can hardly improve matching performance due to the lack of a cooperation mechanism. Therefore, we propose to learn cooperative feature descriptors in a single model by using different losses for supervision. We also propose an orthogonal loss that forces the learned descriptors to be in complementary feature subspaces, to maximize the overall performance.

Unlike naive ensemble strategies that simply combine off-the-shelf descriptors from different models without coordination, our method jointly learns two descriptors within a single network with explicit orthogonal constraint, ensuring complementarity while maintaining efficiency. The choice of two branches is motivated by the observation that a single descriptor space often fails to capture both global and local discriminative cues simultaneously, while more than two branches would introduce diminishing returns with increased computational cost.

**Figure 1:**
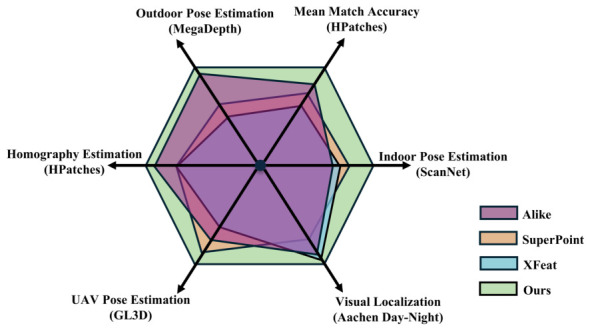
Performance comparison on five public benchmarks. Our method consistently outperforms other sparse matching methods on the tasks of relative pose estimation (ScanNet, MegaDepth, GL3D), visual localization (Aachen Day-Night) and homography estimation (HPatches).

Figure 1 presents a performance overview of the proposed method with benchmark results against leading sparse matching algorithms with respect to four evaluation protocols on five large public datasets: (1) relative pose estimation on ScanNet [12] (indoor), MegaDepth [13] (outdoor) and GL3D [14] (unmanned aerial vehicle, UAV), (2) visual localization on Aachen Day-Night [15], (3) direct matching accuracy test and (4) homography estimation on HPatches [16]. The proposed method consistently outperforms the state-of-the-art sparse matching algorithms across all scenarios, demonstrating superior performance and strong generalization capabilities. More results can be found in Section 4.

The contributions of this paper are as follows:

- A novel complementary local feature description paradigm. Unlike existing sparse methods that learn only a single descriptor per keypoint, we propose to simultaneously learn two cooperative descriptors within a single lightweight CNN. Using two complementary subspaces captures diverse visual characteristics for robust matching, providing a simple yet effective trade-off between model complexity and representational capacity.
- An orthogonal loss for enforcing descriptor complementarity. To prevent redundancy and maximize combined performance, we introduce a novel orthogonal loss. To the best of our knowledge, this is the first work to introduce explicit subspace separation for local feature descriptors, enabling robust matching by jointly reasoning over two similarity matrices.
- State-of-the-art performance and strong generalization. The proposed method achieves leading results on five large-scale benchmarks, consistently outperforming existing sparse matchers (e.g., SuperPoint, XFeat, Alike) and achieves comparable results with global matchers like LoFTR and SuperGlue in several settings.

## 2. Related Work

Learning-based sparse local feature matching methods [5,6,8,9,10] typically involve three steps, namely feature detection, feature description, and feature matching. Early studies [11,17,18,19] only focused on either feature detection or feature description. In terms of feature detection, TILDE [19] uses SIFT [20] to extract repeatable points as labels for learning from the same scene under different illumination conditions and performs better than SIFT on the evaluation dataset. Quad-Network [21] adopts a ranking loss to rank points in a transformation-invariant manner. Then, keypoints are extracted from the top/bottom quantiles of this ranking for unsupervised training. KeyNet [17] combines handcrafted and convolutional neural network features simultaneously to detect keypoints at multiple scales.

In the aspect of feature description, early feature description methods [22,23] are based on image patch learning. Corresponding local descriptors are extracted from the input image patches and similarities are calculated. MatchNet [22] uses cross-entropy loss to train descriptors. L2-Net [23] proposes a progressive sampling strategy for triplet sampling. RF-Net [24] proposes a neighborhood mask to enhance the stability of descriptor training. LIFT [25] imitates SIFT by detecting keypoints, estimating their orientations and employing different neural networks to extract descriptors. HardNet [26] and SOSNet [27] introduce the hardest negative triplets and the second-order similarity of descriptors.

Nevertheless, the receptive field of image-patch-based descriptors is usually limited to specific image regions. In contrast, dense descriptors incorporate a more abundant and comprehensive image context, thereby potentially capturing a broader range of visual information and characteristics. Therefore, in subsequent research, many methods [11,18,28] extract dense descriptors of the entire image through fully convolutional neural networks and integrate the detector into the same network.

SuperPoint [5] designs a self-supervised paradigm and adopts a bootstrapping training strategy to train the model to detect keypoints and jointly train its descriptors using the hinge loss. R2D2 [10] deploys effective loss functions to consider the repeatability and reliability of keypoint detection. D2Net [9] and ASLFeat [29] adopt a similar description and detection paradigm; they first extract dense descriptors and then detect keypoints from the dense descriptors through specific rules. HDD-Net [30] weights the features with the softargmax scores in the grid to train the detector and descriptors simultaneously. In addition, DISK [31] and Reinforced SP [32] relax the keypoint detection and descriptor matching into a probabilistic process and train the network through reinforcement learning. CNDesc [11] adopts a special cross-normalization instead of L2 normalization, and CAPS [18] proposes to learn descriptors from the weak supervision of camera pose. The subsequent PosFeat [33], following the weak supervision approach of CAPS, proposes a line-to-window search strategy. Since weak supervision cannot distinguish the losses caused by the detection and description steps, it decouples the training of the descriptors and the detector. Alike [6] puts forward a partially differentiable keypoint detection module. Building upon this, Aliked [34] incorporates deformable convolutions and derives deformable descriptors. SFD2 [28] implicitly incorporates semantics into the detection and description processes during training. SILK [35] re-evaluates the elements of learning feature extraction and proposes an effective and simple keypoint and descriptor learning strategy. XFeat [8] introduces a lightweight and accurate architecture and provides both sparse and dense matching options.

As far as we know, most existing sparse local feature matching methods often only learn a single low-resolution descriptor map, which greatly limits their applications in complex scenarios. In order to alleviate this problem, we propose a Siamese Orthogonal Descriptor network. It simultaneously learns two complementary descriptor maps within a single CNN and establishes local feature matchings between image pairs in different descriptor sub-spaces respectively. By jointly considering the similarity matrices of the two complementary descriptor maps, more robust matching results are obtained. Meanwhile, following the approach of sparse local feature matching, we design a detector network and train it through distillation.

## 3. Method

Learning-based image matching algorithms are highly dependent on the discriminative power of feature descriptors. While conventional sparse local features achieve high computational efficiency, their matching performance is limited by insufficient discriminative capability. This limitation stems partly from the fact that the matching criterion is encapsulated solely in a single floating-point vector, which struggles to adapt to infinitely complex real-world scenes. In this paper, we propose learning a pair of complementary feature descriptors to address the aforementioned challenges. While following the sparse feature learning pipeline to preserve overall efficiency, we augment each keypoint with multiple descriptors that are jointly learned in a Siamese CNN architecture. To effectively coordinate these descriptors and maximize their collective performance, we introduce an orthogonal loss. Finally, robust matching results between input images can be obtained by jointly considering the feature similarities with respect to the two sets of descriptors.

The remainder of this section is organized as follows. Section 3.1 details the network architecture. Next, Section 3.2 presents the supervision strategies for learning different local features, followed by the complementary feature learning method introduced in Section 3.3. For the completeness of the algorithm, a feature detection module is also learned, as described in Section 3.4. Finally, Section 3.5 outlines the inference logic.

### 3.1. Overall Framework

**Figure 2:**
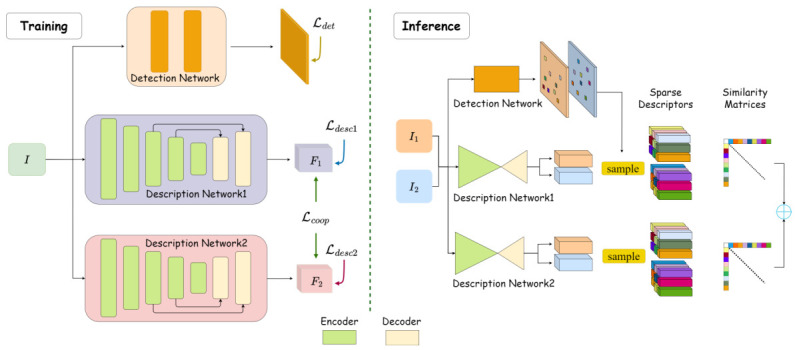
Overall framework. Two descriptor branches are supervised by different losses ($L_{desc1}$, $L_{desc2}$) and coordinated by an orthogonal loss $L_{coop}$. A keypoint detector is trained via distillation ($L_{det}$). During inference, keypoints are detected from the score map, and matching is performed by a weighted sum of two similarity matrices.

The overall architecture of the proposed model is illustrated in Figure 2. The network consists of three branches: one for keypoint localization and two for descriptor learning. Each descriptor branch consists of an encoder and a decoder. The encoder is composed of five residual modules, where the feature resolution of the *n*-th module is ${½}_{n-1}$ of the input size, and the number of feature channels is $2^{n+3}$. The decoder employs two upsampling layers to restore the feature resolution to $\frac{1}{4}$ of the original input.

The model takes a single image of size H×W as input and outputs two descriptor maps, $F_{1}$ and $F_{2}$, each of shape $\frac{H}{4}\times \frac{W}{4}\times 128$. The output descriptors $F_{1}$ and $F_{2}$ will be L2-normalized before being used. In addition, a score map S∈(0,1) of shape H×W×1 is learned for keypoint detection.

During inference, we apply non-maximum suppression (NMS) to *S* to detect pixel-level keypoints $P=\left\{{\left[x,y\right]}^{T}\right\}$. The corresponding sparse feature descriptors $D_{1}=F_{1}\left(P\right)=\left\{d_{1}\in R^{128}\right\}$ and $D_{2}=F_{2}\left(P\right)=\left\{d_{2}\in R^{128}\right\}$ are then retrieved from $F_{1}$ and $F_{2}$ via bilinear interpolation.

### 3.2. Descriptor Learning

The model is trained using different losses as shown in Figure 2. The two descriptors predicted by the model are designed to be complementary while being different. Thus they are supervised with different losses in model learning. Specifically, we use the negative log-likelihood (NLL) loss [8] to train the first descriptor branch because it encourages soft assignment of correspondences through bidirectional softmax normalization, which tends to produce more globally consistent matching distributions. The hardest negative triplet (HNT) loss [26,36] is chosen to train the second descriptor branch as it focuses on local discrimination by explicitly pushing apart the most confusing negative pairs. This combination allows one descriptor to excel at global matching context while the other specializes in fine-grained local discrimination.

We supervise and train our descriptor maps $D_{1}$ and $D_{2}$ with pixel correspondences. Given a pair of training images (A,B) with a set of point matches $M_{A,B}=\left\{\left(a_{i}\in A,b_{j}\in B\right)\right\}$, we firstly sample sparse point descriptors in $F_{1},F_{2}$ to get $D_{1},D_{2}$. Then the cosine similarities are computed as $C_{1}=D_{1}\left(A\right)D_{1}^{T}\left(B\right)$ and $C_{2}=D_{2}\left(A\right)D_{2}^{T}\left(B\right)$.

For the first branch, the NLL loss is applied to the forward and backward matching probabilities of every ground-truth point correspondence respectively. For each pair $\left(a_{i},b_{j}\right)\in M_{A,B}$, the forward matching probability is the similarity distribution of matching $a_{i}\in A$ with every point in *B*, which is computed by a row-wise softmax operation to $C_{1}$. The backward matching probability is analogously defined as the distribution of matching $b_{j}\in B$ with every point in *A*, and can be computed via a column-wise softmax to $C_{1}$. Then the loss is defined as the sum of them:

(1) $$L_{desc1}=-\frac{1}{|M_{A,B}|}\sum_{\left(i,j\right)\in M_{A,B}}\left(\log\left(p_{i,i}\right)+\log\left(p_{j,j}^{\prime }\right)\right)$$

where $p_{i,i}$ is the *i*-th diagonal element of the row softmax result of $C_{1}$, and $p_{j,j}^{\prime }$ is the *j*-th diagonal element of the column softmax result of $C_{1}$.

For the second descriptor branch, the HNT loss is employed for training supervision. The distance between a pair of descriptors is defined as:

(2) $$dist\left(d_{i},d_{j}\right)={∥d_{i}-d_{j}∥}^{2}$$

It can be easily seen that $dist\left(d_{i},d_{j}\right)=2-d_{i}^{T}d_{j}$. Thus the pair-wise descriptor distance can be retrieved from the similarity matrix $C_{2}$.

We then define the loss of the second descriptor branch as:

(3) $$L_{desc2}=\frac{1}{|M_{A,B}|}\sum_{\left(i,j\right)\in M_{A,B}}\max\left(0,dist\left(d_{a_{i}},d_{b_{j}}\right)-\min_{k\neq j}dist(d_{a_{i}},d_{b_{k}})+m\right)$$

where *m* is the margin parameter. The loss is zero only when the distance between the positive pair is smaller than that of the hardest negative pair by a certain margin.

### 3.3. Cooperative Descriptor Learning

For the learned two descriptors, we expect to maximize their combined performance. Therefore, we introduce an orthogonal loss to make them mutually decoupled but complementary to each other. The core idea is to constrain the embedding spaces of different descriptors to be orthogonal to each other.

We introduce the orthogonal loss to learn cooperative descriptors. The two descriptor branches are designed to be symmetric, and any image position will correspond to two structurally identical feature description vectors. We thus define the loss as the including angle between the vectors. Then the cooperative loss of the network is defined as the mean angles of all the keypoints.

The cooperative learning loss $L_{coop}$ is defined as:

(4) $$L_{coop}=\frac{1}{2|M_{A,B}|}\sum_{\left(i,j\right)\in M_{A,B}}\left(\left|\langle d_{1,a_{i}},d_{2,a_{i}}\rangle \right|+\left|\langle d_{1,b_{j}},d_{2,b_{j}}\rangle \right|\right)$$

### 3.4. Keypoint Localization

A keypoint detector is simultaneously learned to identify repetitive and reliable local regions in images. However, due to the ambiguity in the definition of keypoints, few datasets come with keypoint labels. Consequently, the training of keypoints is rather challenging. Currently, most methods for training local feature detectors either leverage epipolar geometry or homography transformation to establish point correspondences and then train the detectors by optimizing the scores of the corresponding keypoints. Nevertheless, this approach typically depends on large datasets and is difficult to train. Therefore, in this work, to simplify the training of keypoints and to obtain good generalization, we train the keypoint detector through distillation of a known keypoint detector.

In the knowledge distillation paradigm, a teacher model guides the training of the keypoint detector, which is called the student model. Both models predict a score map for the input image, from which keypoints are localized by detecting peaks and applying a threshold. Here the standard binary cross-entropy (BCE) loss is employed to guide the training of the keypoint detector.

(5) $$L_{det}=BCE\left(S,S^{\prime }\right)$$

where *S* and $S^{\prime }$ are the predicted score maps of the proposed model and the teacher model respectively. They are passed to the Sigmoid activation to limit the range in (0,1).

The final loss L is then a linear combination of all losses:

(6) $$L=\alpha L_{desc1}+\beta L_{desc2}+\gamma L_{coop}+\eta L_{det}$$

where {α,β,γ,η} are hyperparameters to adjust the magnitude of the different losses.

### 3.5. Inference

The orthogonal training method pushes the two descriptors to be learned in different subspaces. In order to effectively utilize these two complementary descriptor maps, we set different weights to combine their similarity matrices, thereby effectively filtering out unreliable matches while retaining correct matches and further establishing robust correspondences.

Final matches between images will be determined by the weighted sum of the similarities with respect to two sets of local descriptors. The overall detection score is defined as:

(7) $$score=\mu \cdot C_{1}+\nu \cdot C_{2}$$

where $C_{1}$ and $C_{2}$ are the cross products between the two descriptor sets. μ and ν are constant coefficients.

## 4. Experiments

**Figure 3:**
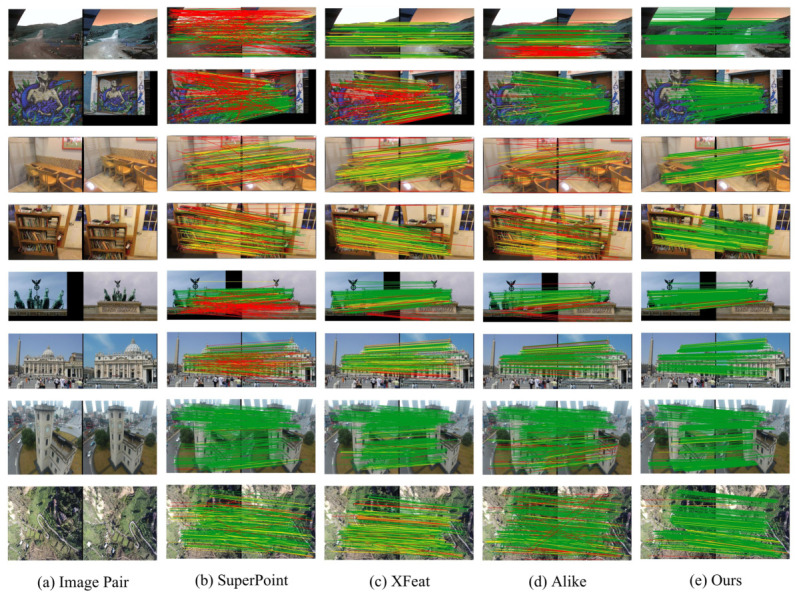
Qualitative matching results on HPatches, ScanNet, MegaDepth, and GL3D (top to bottom). Our method produces more correct matches (green) and fewer incorrect matches (red) compared to SuperPoint, XFeat, and Alike.

The proposed method generates a pair of complementary descriptors for each keypoint, enabling robust feature matching under various challenging conditions. A preliminary performance overview is provided in Figure 3, where raw matches from our method and three state-of-the-art approaches are visualized on representative image pairs from the HPatches [16] (planar scenes), ScanNet [12] (indoor), MegaDepth [13] (outdoor), and GL3D [14] (aerial) datasets. For evaluation, matches are color-coded based on reprojection error: green for correct matches (error < 5 pixels), yellow for inaccurate matches (error between 5–10 pixels), and red for incorrect matches (error > 10 pixels). As shown, our method consistently produces more correct matches across diverse scenarios. The remainder of this section presents detailed evaluations on multiple downstream tasks including homography estimation, pose estimation, and visual localization across multiple datasets.

### 4.1. Implementation Details

The proposed method is implemented in PyTorch 2.8 on a RTX 4090 GPU. Adhering to the experimental setup of XFeat [8], we perform a mixed training on the Megadepth [13] and COCO datasets [37] with a sample ratio of 5:2. Input images are resized to 640×480 and the batch size is set to 7. We optimize the model using the Adam optimizer with a learning rate of $3\times 10^{-4}$. Our keypoint detector is trained under the guidance of XFeat as the teacher network. The loss coefficients are set as α=1, β=0.1, γ=2 and η=1. Following [9], the margin param *m* is set to 1. In the reference stage, we assign μ=0.75 and ν=0.25 to the corresponding similarity scores.

### 4.2. Homography Estimation

We evaluate our method on the widely adopted HPatches dataset [16] for homography estimation. HPatches is designed to benchmark local image descriptors under varying viewpoint and illumination conditions. It comprises 108 sequences, each containing one reference image and five images transformed by ground-truth homographies.

**Evaluation protocol.** We adopt two established metrics for evaluation on HPatches. The first is homography estimation accuracy, which measures the alignment error between the estimated homography and the ground-truth transformation from the reference to each target image. Accuracy is reported as the percentage of correctly reprojected image corners under error thresholds of 1, 3 and 5 pixels. The second metric is the mean matching accuracy (MMA), defined as the proportion of correctly matched points among all predicted correspondences.

**Baselines.** We compare our approach with a range of feature matching algorithms, including sparse local feature matchers such as SuperPoint (SP) [5], Alike [6], R2D2 [10], D2-Net [9], SILK [35], DISK [31], XFeat [8], ASLFeat [29], CAPS [18], and ISRF [38]. We also include global matching methods that necessitate two images as network input to predict feature correspondences, like Patch2Pix [39], LoFTR [40], and SuperGlue [41]. SuperGlue is a learnable middle-end matcher that augments matching with an attentional graph neural network. Here, we pair it with SuperPoint as the front-end feature extractor. For all baseline methods, we use the pretrained models provided by their authors. Specifically, for XFeat, we evaluate only its sparse matching variant, and for Alike, we assess the Alike-N model.

**Results.** The comparison results are presented in Table 1. With the exception of Patch2Pix, which takes two frames as input and directly predicts matches by complex multi-scale matching refinement, all other competing methods follow the sparse local feature matching paradigm. For a fair comparison, we apply the mutual nearest neighbor (MNN) criterion to establish matches and use the same RANSAC procedure for homography estimation.

**Table 1:** Homography estimation accuracy on HPatches (corner error thresholds: 1, 3, 5 pixels). MNN is used for sparse matching except for Patch2Pix. Bottom rows show results when replacing the detector with SuperPoint (SP). Best results are in bold.

Method	Overall	Illumination	Viewpoint	Matches
	**Accuracy** (%, τ≤1/3/5 **px**)			
SuperPoint [5]+MNN	0.50/0.82/0.89	0.63/0.95/**0.99**	0.39/0.71/0.81	1.1 K
D2Net [9]+MNN	0.40/0.75/0.85	0.65/0.95/**0.99**	0.16/0.56/0.71	2.2 K
R2D2 [10]+MNN	0.45/0.72/0.82	0.72/0.95/**0.99**	0.20/0.50/0.65	1.1 K
XFeat [8]+MNN	0.50/0.81/0.90	0.69/0.95/0.98	0.33/0.68/0.82	2.0 K
Alike [6]+MNN	0.51/0.81/0.89	0.65/0.94/0.98	0.39/0.69/0.81	1.2 K
DISK [31]+MNN	0.54/0.82/0.89	0.66/0.94/**0.99**	**0.43**/0.71/0.80	2.3 K
Patch2Pix [39]	**0.58**/0.85/0.91	**0.75**/0.97/**0.99**	0.41/0.74/0.84	1.3 K
Ours+MNN	0.53/**0.87**/**0.92**	0.70/**0.98**/**0.99**	0.37/**0.77**/**0.85**	2.7 K
SP+CAPS [18]+MNN	0.53/0.83/0.90	**0.68**/0.96/0.99	0.40/0.72/0.82	1.1 K
SP+XFeat+MNN	0.51/0.82/0.90	0.64/0.95/0.99	0.39/0.70/0.82	1.1 K
SP+Alike+MNN	0.54/0.84/0.90	0.65/0.96/0.99	0.44/0.73/0.82	1.1 K
SP+Ours+MNN	**0.59**/**0.87**/**0.93**	0.67/**0.98**/**1.00**	**0.50**/**0.77**/**0.86**	1.1 K

As shown in the table, our method achieves the best performance under both illumination and viewpoint changes among all sparse matching approaches. Although DISK operates on full-resolution feature maps for descriptor learning, it is consistently outperformed by our method in most settings. Although we learn feature descriptors at a 1/4 resolution, our descriptors demonstrate a stronger discrimination power.

Furthermore, an additional experiment in which we replace our keypoint detector with SuperPoint is shown in the last row of the table. The results indicate that our distilled lightweight detector attains performance on par with state-of-the-art detectors while predicting a significantly larger number of keypoints.

**Figure 4:**
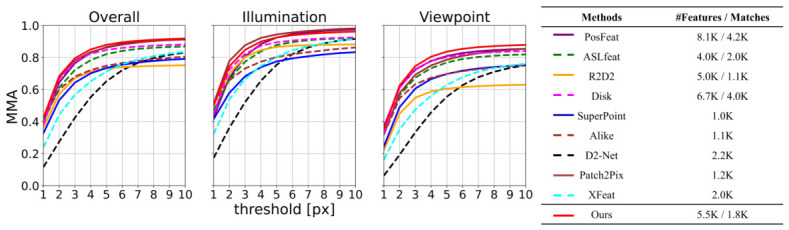
Mean matching accuracy (MMA) on HPatches. Our method achieves the highest accuracy across all pixel thresholds (1 to 10). The legend shows the average number of keypoints and matches per method.

Evaluation results of mean matching accuracy on HPatches dataset are plotted in Figure 4 where a higher curve means better performance. We report the mean matching accuracy (MMA) of different methods under the pixel threshold ranging from 1 to 10. In the legend we report the average number of the detected keypoints and of the final matches. As shown, the proposed method is able to detect a larger number of keypoints while consistently establishing more reliable correspondences under varying challenges.

### 4.3. Relative Pose Estimation

Vision-based relative pose estimation is typically performed by estimating the rotational and translational parameters between a reference image and a novel view. Here the accuracy of relative pose estimation is estimated on the MegaDepth [13], ScanNet [12] and GL3D [14] datasets. We utilize sparse local feature matchers to generate point correspondences between two images and adopt a same RANSAC-based PnP method for relative pose estimation.

**Datasets.** Three datasets are used here. The first is the MegaDepth dataset which comprises 196 outdoor scenes with depth maps and sparse 3D reconstruction results. Following the evaluation protocol in LoFTR [40], we extract 1500 image pairs and resize them to a maximum dimension of 1200 pixels for a fair comparison. The second is the ScanNet dataset which consists of 1613 indoor sequences with wide baselines and extensive textureless regions. Following LOFTR, we resize all images and depth maps to 640×480. The third one is the GL3D dataset which contains 125,623 high-resolution drone-view images covering 543 different scenes. For our experiments, 6722 image pairs with an overlap ratio between 0.5 and 0.6 are selected and resized to 1200×1200 pixels for evaluation.

**Evaluation protocol.** We adopt the widely used pose AUC metric [40] to measure the pose estimation accuracy. For a given image pair, the pose error is defined as the maximum angular error between the estimated rotation and translation matrices and the groundtruths. The overall performance is then reported as the AUC (Area Under the Curve) of the pose error under thresholds $\left(5^{\circ },10^{\circ },20^{\circ }\right)$.

**Results.** We compare the proposed method with state-of-the-art approaches on relative pose estimation across three datasets. The results are shown in Table 2. For each method, a single pretrained model is used for evaluation without any fine-tuning. Local image matchers predict sparse keypoints and descriptors from a single image, making them more efficient but generally lag behind global matchers in accuracy, as the latter leverage two images and global context for reasoning. Our method consistently outperforms all traditional local matching methods and achieves competitive results with global approaches. Notably, despite being trained solely on ground-view images, it generalizes effectively to the aerial GL3D dataset, demonstrating strong cross-domain robustness. Despite its superiority among local matchers, our method still struggles in textureless scenarios. Quantitatively, the performance gap between our method and LoFTR (a semi-dense global matcher) varies across datasets: 34.8% on ScanNet (indoor, textureless), 26.6% on MegaDepth (outdoor, medium texture), and only 0.7% on GL3D (drone view, rich texture), confirming that texture deficiency is the primary failure mode of local feature matching.

### 4.4. Visual Localization

We evaluate the visual localization accuracy on the Aachen Day-Night dataset [15]. A positional error threshold and a directional error threshold are simultaneously applied to define a successful recall. In accordance with the official evaluation pipeline, three sets of thresholds are adopted, namely (0.5 m, $2^{\circ })$, (1 m, $5^{\circ })$ and (5 m, $10^{\circ })$.

**Results.**Table 3 reports the visual localization accuracy on the Aachen Day-Night dataset, comparing seven local and three global matching methods. Notably, only R2D2 and ISRF among these baselines have been trained on the Aachen dataset. Our method achieves superior or comparable results to state-of-the-art local and global matchers (e.g., SuperGlue, LoFTR) in most settings, demonstrating strong generalization capability.

### 4.5. Ablation Study

**Directly combining descriptors from different models.** Here, we conduct an ablation study to investigate whether directly combining descriptors from different models can improve matching performance. The results are shown in Table 4. Three local image matching models are evaluated: SuperPoint, XFeat, and Alike. We report pose estimation accuracy on the ScanNet dataset. No apparent performance gain is observed when combining descriptors from SuperPoint and XFeat. Only Alike shows improvement after combining with XFeat, but the resulting performance is merely on par with using XFeat alone. The experiment indicates that directly combining different descriptors without a comprehensive learning mechanism can hardly improve image matching performance.

**Effects of different descriptor learning strategies.** Recent feature matching researches generally adopt either the Negative Log-Likelihood (NLL) loss [8] or the Hardest Negative Triplet (HNT) loss [36] for descriptor learning. The NLL loss encourages soft, globally consistent matching distributions through bidirectional softmax normalization, making it suitable for capturing global context. The HNT loss focuses on local discrimination by explicitly pushing apart the most confusing negative pairs, making it suitable for fine-grained feature discrimination. Here, we evaluate different loss configurations for descriptor learning on the MegaDepth pose estimation task, with results reported in Table 5. Using two complementary descriptors significantly improves matching accuracy over a single descriptor. Furthermore, employing different losses for the two branches (NLL for one, HNT for the other) yields the best overall performance.

**Figure 5:**
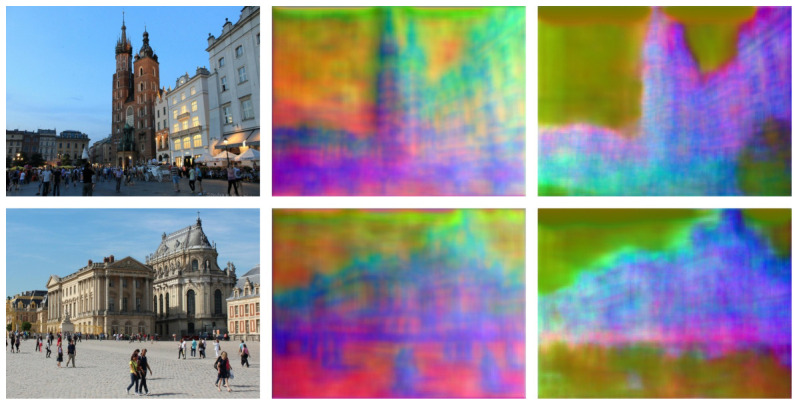
Visualization of descriptor complementarity using PCA. Descriptor 1 (middle) responds strongly to edge-rich regions, while Descriptor 2 (right) focuses on texture areas, confirming the effect of orthogonal loss.

Visualization of descriptor complementarity using PCA is shown in Figure 5. For each input image, we project the 128-dimensional descriptor vectors onto the first three principal components and map them to RGB channels. It can be seen that Descriptor 1 responds strongly to contours and edges, while Descriptor 2 shows more uniform activation over textured regions, confirming the complementary behavior enforced by the orthogonal loss.

**Effectiveness of the complementary descriptors.** Though the proposed method learns a keypoint detector through distillation, effectiveness of the proposed complementary descriptors can be separately analyzed by replacing the description module of existing sparse local feature matchers. Refer to Table 6 for details. We replace the descriptors of SuperPoint [5], Alike [6] and SILK [35] while retaining their keypoint detectors to test the matching accuracy on the MegaDepth dataset. Significant performance gains are observed in the experiments, verifying the effectiveness of our learned descriptors.

### 4.6. Running Efficiency

Feature matching serves as a fundamental building block in many real-time visual computing systems, making model inference efficiency a critical evaluation metric. We compare the mean inference time (MIT) and computational complexity (in GFLOPs) of different image matching algorithms by performing a forward pass of the pretrained models using randomly sampled images of size 640×480. All experiments are conducted on the same computer equipped with an AMD Ryzen 9 5950X CPU (16 cores, 3.4 GHz), 64 GB RAM and an NVIDIA RTX 4090 GPU, with both data and parameters in float32 format.

**Results.** The computational complexity and inference time of each model are reported in Table 7. For Patch2Pix, a batch size of 8 is used instead of 16 to avoid GPU out-of-memory errors. For SuperGlue, the number of keypoints is fixed to 1024. Certain local matching methods, such as R2D2, PoSFeat, SILK, and DISK, operate at full image scale and consequently incur substantial computational overhead. In contrast, other local feature matchers are significantly more efficient than global ones in terms of both model size and inference speed. The proposed algorithm achieves a high inference speed for both batch-1 and batch-16 tests.

## 5. Conclusions

In this work, we design a cooperative descriptor learning model that enhances image matching performance with minimal computational overhead. An orthogonal loss is introduced to enforce complementary subspace learning for feature descriptors. Extensive experiments demonstrate superior performance over existing approaches in both matching accuracy and model generalization, while maintaining leading runtime efficiency.

**Limitations and future work.** Despite its effectiveness, our method has several limitations. First, the orthogonal loss enforces complementarity globally across the entire image, which may not be optimal for local regions where both descriptors struggle (e.g., highly textureless areas). Second, our keypoint detector is distilled from XFeat, inheriting its potential biases. Third, as shown in Table 2, the method remains vulnerable to textureless scenes (e.g., ScanNet) where repeatable keypoints are inherently difficult to extract. Future work will focus on region-adaptive complementarity and integration with semi-dense or dense matching paradigms to address these limitations.

## Figures and Tables

**Table 2:** Pose estimation AUC (%) on MegaDepth (outdoor), ScanNet (indoor), and GL3D (aerial). Best results among local and global methods are in bold. Our method consistently outperforms all local matchers and generalizes well to the aerial domain despite being trained only on ground-view images.

Pose Estimation AUC										
Category	Method	MegaDepth			ScanNet			GL3D		
		@5°	@10°	@20°	@5°	@10°	@20°	@5°	@10°	@20°
Local	SuperPoint [5]	28.73	44.29	58.26	7.77	18.67	31.64	60.61	68.61	73.43
	SILK [35]	29.33	40.96	52.05	7.25	16.45	27.92	60.48	68.26	72.77
	XFeat [8]	26.82	43.76	59.04	6.84	17.49	31.1	53.5	64.09	70.75
	D2Net [9]	17.87	32.11	48.51	3.19	10.29	22.15	50.82	62.15	69.57
	R2D2 [10]	27.27	40.95	52.74	-	-	-	-	-	-
	Alike [6]	38.36	54.62	67.88	4.73	10.76	18.09	59.72	67.81	72.63
	DISK [31]	-	-	-	8.51	18.64	31.27	61.52	69.05	73.57
	Ours	**39.57**	**55.30**	**68.39**	**11.52**	**26.11**	**42.13**	**63.45**	**70.6**	**74.71**
Global	Patch2Pix [39]	40.76	55.74	67.63	-	-	-	-	-	-
	SP+SuperGlue [41]	45.93	63.37	76.97	14.61	30.95	47.41	**64.56**	**72.27**	**76.72**
	LoFTR [40]	**53.05**	**69.77**	**81.68**	**17.54**	**34.30**	**50.92**	64.34	70.96	74.74

**Table 3:** Visual localization accuracy on Aachen Day-Night v1 and v1.1 (pose error thresholds: (0.5 m, 2°), (1 m, 5°), (5 m, 10°)). Best and second-best results are in bold and underlined, respectively.

Category	Method	Aachen Day-Night v1			Aachen Day-Night v1.1		
		**(0.5 m, 2°)**	**(1 m, 5°)**	**(5 m, 10°)**	**(0.5 m, 2°)**	**(1 m, 5°)**	**(5 m, 10°)**
Local	SuperPoint [5]	77.6	85.7	95.9	-	-	-
	D2Net [9]	74.5	86.7	**100**	-	-	-
	R2D2 [10]	76.5	**90.8**	**100**	71.2	86.9	97.9
	ASLFeat [29]	77.6	89.8	**100**	72.3	86.4	97.9
	ISRF [38]	-	-	-	69.1	87.4	98.4
	Alike [6]	**81.6**	88.8	99	-	-	-
	XFeat [8]	77.6	89.8	98.0	-	-	-
	Ours	80.6	**90.8**	**100**	**73.3**	86.9	98.4
Global	Patch2Pix [39]	79.6	87.8	**100**	-	-	-
	SP+SperGlue [41]	79.6	**90.8**	**100**	**73.3**	88.0	98.4
	LoFTR [40]	-	-	-	72.8	**88.5**	**99.0**

**Table 4:** Ablation study on directly combining descriptors from different models (ScanNet pose estimation AUC). No consistent improvement is observed, demonstrating that naive ensemble without cooperative learning is ineffective.

Method	Pose Estimation AUC		
	@5°	@10°	@20°
SuperPoint [5]	7.77	18.67	31.64
XFeat [8]	6.84	17.49	31.1
Alike [6]	4.73	10.76	18.09
SuperPoint+XFeat	7.7	18.79	33.32
SuperPoint+Alike	8.67	19.45	31.96
XFeat+Alike	7.73	18.19	31.76

**Table 5:** Ablation study on descriptor learning losses (MegaDepth pose estimation AUC). Using two complementary losses (HNT + NLL) achieves the best performance, outperforming single-loss or same-loss configurations.

Training Loss		Pose Estimation AUC		
$L_{\mathrm{desc}1}$	$L_{\mathrm{desc}2}$	**@5°**	**@10°**	**@20°**
HNT	/	37.3	52.96	66.54
/	NLL	36.91	52.59	66.0
HNT	HNT	37.75	53.33	66.75
NLL	NLL	38.4	53.42	66.77
HNT	NLL	39.57	55.3	68.39

**Table 6:** Generalization of our descriptors. Replacing the descriptors of SuperPoint, SILK, and Alike with our complementary descriptors significantly improves pose estimation AUC on MegaDepth, demonstrating their strong discriminative power.

Method		Pose Estimation AUC		
**Detector**	**Descriptor**	**@5°**	**@10°**	**@20°**
SuperPoint	SuperPoint	28.73	44.29	58.26
	Ours	37.61	54.24	67.94
SILK	SILK	29.33	40.96	52.05
	Ours	36.8	52.94	66.93
Alike	Alike	38.36	54.62	67.88
	Ours	39.69	55.17	68.06

**Table 7:** Model complexity and mean inference time (MIT) at batch sizes 1 and 16 for different matching algorithms. The best and second-best results are highlighted in bold and underlined respectively. Our method achieves strong parallelism and real-time efficiency.

Category	Method	Size (MB)	GFLOPs	MIT@1 (ms)	MIT@16 (ms)
Local	SuperPoint	1.3	26	5	57
	D2Net	7.6	85	6	80
	R2D2	0.5	149	15	212
	Alike	**0.3**	8	21	69
	XFeat	0.7	**1.3**	6	**19**
	PoSFeat	21.1	209	17	203
	SILK	0.9	275	25	329
	DISK	1.1	99	34	131
	Ours	4.8	29	**3**	46
Global	Patch2Pix	17.6	416	106	807
	SperGlue	11.5	23	9	112
	LoFTR	11.0	325	38	523

## Data Availability

The source code of the proposed algorithm is publicly available at https://github.com/FYL0123/BSD (accessed on 13 April 2026).

## References

[1] Zhan Z., Yu Y., Xia R., Gan W., Xie H., Perda G., Morelli L., Remondino F., Wang X. (2025). SfM on-the-fly: A robust near real-time SfM for spatiotemporally disordered high-resolution imagery from multiple agents. ISPRS J. Photogramm. Remote Sens..

[2] Hu X., Wu Y., Zhao M., Yang L., Zhang X., Ji X. (2025). PAS-SLAM: A Visual SLAM System for Planar-Ambiguous Scenes. IEEE Trans. Circuits Syst. Video Technol..

[3] Chen K., Xiao J., Liu J., Tong Q., Zhang H., Liu R., Zhang J., Ajoudani A., Chen S. (2025). Semantic Visual Simultaneous Localization and Mapping: A Survey. IEEE Trans. Intell. Transp. Syst..

[4] Lømo T., Torresen J., Kolberg M., Maffei R. (2025). Multi Map Visual Localization for Unmanned Aerial Vehicles. IEEE Robot. Autom. Lett..

[5] DeTone D., Malisiewicz T., Rabinovich A. SuperPoint: Self-Supervised Interest Point Detection and Description. Proceedings of the Conference on Computer Vision and Pattern Recognition Workshops.

[6] Zhao X., Wu X., Miao J., Chen W., Chen P.C.Y., Li Z. (2023). ALIKE: Accurate and Lightweight Keypoint Detection and Descriptor Extraction. IEEE Trans. Multimed..

[7] Edstedt J., Sun Q., Bökman G., Wadenbäck M., Felsberg M. RoMa: Robust Dense Feature Matching. Proceedings of the IEEE/CVF Conference on Computer Vision and Pattern Recognition.

[8] Potje G., Cadar F., Araujo A., Martins R., Nascimento E.R. XFeat: Accelerated Features for Lightweight Image Matching. Proceedings of the IEEE/CVF Conference on Computer Vision and Pattern Recognition.

[9] Dusmanu M., Rocco I., Pajdla T., Pollefeys M., Sivic J., Torii A., Sattler T. D2-Net: A Trainable CNN for Joint Description and Detection of Local Features. Proceedings of the IEEE/CVF Conference on Computer Vision and Pattern Recognition.

[10] Revaud J., De Souza C., Humenberger M., Weinzaepfel P. R2D2: Reliable and Repeatable Detector and Descriptor. Proceedings of the Neural Information Processing Systems (NeurIPS).

[11] Wang C., Xu R., Xu S., Meng W., Zhang X. (2023). CNDesc: Cross Normalization for Local Descriptors Learning. IEEE Trans. Multimed..

[12] Dai A., Chang A.X., Savva M., Halber M., Funkhouser T., Nießner M. ScanNet: Richly-Annotated 3D Reconstructions of Indoor Scenes. Proceedings of the IEEE/CVF Conference on Computer Vision and Pattern Recognition.

[13] Li Z., Snavely N. MegaDepth: Learning Single-View Depth Prediction from Internet Photos. Proceedings of the IEEE/CVF Conference on Computer Vision and Pattern Recognition.

[14] Shen T., Luo Z., Zhou L., Zhang R., Zhu S., Fang T., Quan L. (2018). Matchable Image Retrieval by Learning from Surface Reconstruction. Proceedings of the Asian Conference on Computer Vision.

[15] Zhang Z., Sattler T., Scaramuzza D. (2021). Reference Pose Generation for Long-Term Visual Localization via Learned Features and View Synthesis. Int. J. Comput. Vis..

[16] Balntas V., Lenc K., Vedaldi A., Mikolajczyk K. HPatches: A Benchmark and Evaluation of Handcrafted and Learned Local Descriptors. Proceedings of the IEEE Conference on Computer Vision and Pattern Recognition.

[17] Barroso-Laguna A., Mikolajczyk K. Key.Net: Keypoint Detection by Handcrafted and Learned CNN Filters Revisited. Proceedings of the IEEE/CVF International Conference on Computer Vision.

[18] Wang Q., Zhou X., Hariharan B., Snavely N. (2020). Learning Feature Descriptors Using Camera Pose Supervision. Proceedings of the European Conference on Computer Vision.

[19] Verdie Y., Yi K., Fua P., Lepetit V. TILDE: A Temporally Invariant Learned Detector. Proceedings of the IEEE Conference on Computer Vision and Pattern Recognition.

[20] Lowe D.G. (2004). Distinctive Image Features from Scale-Invariant Keypoints. Int. J. Comput. Vis..

[21] Savinov N., Seki A., Ladicky L., Sattler T., Pollefeys M. Quad-Networks: Unsupervised Learning to Rank for Interest Point Detection. Proceedings of the IEEE Conference on Computer Vision and Pattern Recognition.

[22] Han X., Leung T., Jia Y., Sukthankar R., Berg A.C. MatchNet: Unifying Feature and Metric Learning for Patch-Based Matching. Proceedings of the IEEE Conference on Computer Vision and Pattern Recognition.

[23] Tian Y., Fan B., Wu F. L2-Net: Deep Learning of Discriminative Patch Descriptor in Euclidean Space. Proceedings of the IEEE Conference on Computer Vision and Pattern Recognition.

[24] Shen X., Wang C., Li X., Yu Z., Li J., Wen C., Cheng M., He Z. RF-Net: An End-To-End Image Matching Network Based on Receptive Field. Proceedings of the IEEE Conference on Computer Vision and Pattern Recognition.

[25] Yi K.M., Trulls E., Lepetit V., Fua P. (2016). LIFT: Learned Invariant Feature Transform. Proceedings of the European Conference on Computer Vision.

[26] Mishchuk A., Mishkin D., Radenovic F., Matas J. (2017). Working Hard to Know Your Neighbor’s Margins: Local Descriptor Learning Loss. NeurIPS.

[27] Tian Y., Yu X., Fan B., Wu F., Heijnen H., Balntas V. SOSNet: Second Order Similarity Regularization for Local Descriptor Learning. Proceedings of the IEEE Conference on Computer Vision and Pattern Recognition.

[28] Xue F., Budvytis I., Cipolla R. SFD2: Semantic-Guided Feature Detection and Description. Proceedings of the IEEE Conference on Computer Vision and Pattern Recognition.

[29] Luo Z., Zhou L., Bai X., Chen H., Zhang J., Yao Y., Li S., Fang T., Quan L. ASLFeat: Learning Local Features of Accurate Shape and Localization. Proceedings of the IEEE Conference on Computer Vision and Pattern Recognition.

[30] Barroso-Laguna A., Verdie Y., Busam B., Mikolajczyk K. HDD-Net: Hybrid Detector Descriptor with Mutual Interactive Learning. Proceedings of the Asian Conference on Computer Vision.

[31] Tyszkiewicz M., Fua P., Trulls E. (2020). DISK: Learning Local Features with Policy Gradient. NeurIPS.

[32] Bhowmik A., Gumhold S., Rother C., Brachmann E. Reinforced Feature Points: Optimizing Feature Detection and Description for a High-Level Task. Proceedings of the IEEE Conference on Computer Vision and Pattern Recognition.

[33] Li K., Wang L., Liu L., Ran Q., Xu K., Guo Y. Decoupling Makes Weakly Supervised Local Feature Better. Proceedings of the IEEE Conference on Computer Vision and Pattern Recognition.

[34] Zhao X., Wu X., Chen W., Chen P.C.Y., Xu Q., Li Z. (2023). ALIKED: A Lighter Keypoint and Descriptor Extraction Network via Deformable Transformation. IEEE Trans. Instrum. Meas..

[35] Gleize P., Wang W., Feiszli M. (2023). SiLK: Simple Learned Keypoints. arXiv.

[36] Potje G., Cadar F., Araujo A., Martins R., Nascimento E.R. Enhancing Deformable Local Features by Jointly Learning to Detect and Describe Keypoints. Proceedings of the IEEE/CVF Conference on Computer Vision and Pattern Recognition.

[37] Lin T.Y., Maire M., Belongie S., Hays J., Perona P., Ramanan D., Dollár P., Zitnick C.L. (2014). Microsoft COCO: Common Objects in Context. Proceedings of the European Conference on Computer Vision.

[38] Melekhov I., Brostow G.J., Kannala J., Turmukhambetov D. (2020). Image Stylization for Robust Features. arXiv.

[39] Zhou Q., Sattler T., Leal-Taixe L. Patch2Pix: Epipolar-Guided Pixel-Level Correspondences. Proceedings of the IEEE Conference on Computer Vision and Pattern Recognition.

[40] Sun J., Shen Z., Wang Y., Bao H., Zhou X. LoFTR: Detector-Free Local Feature Matching with Transformers. Proceedings of the IEEE Conference on Computer Vision and Pattern Recognition.

[41] Sarlin P.E., DeTone D., Malisiewicz T., Rabinovich A. SuperGlue: Learning Feature Matching with Graph Neural Networks. Proceedings of the IEEE Conference on Computer Vision and Pattern Recognition.

